# Membrane fixation for osseous graft stabilization in periodontally accelerated osteogenic orthodontics: a comparative study

**DOI:** 10.1186/s12903-019-0964-5

**Published:** 2020-01-28

**Authors:** Xiaohan Liu, Baoting Fan, Ahmed Abdelrehem, Zhigui Ma, Chi Yang

**Affiliations:** 1 Department of Oral Surgery, Ninth People’s Hospital, Shanghai Jiao Tong University School of Medicine. Shanghai Key Laboratory of Stomatology & Shanghai Research Institute of Stomatology. National Clinical Research Center of Stomatology, 639 Zhizaoju Road, Shanghai, 200011 China; 2 Department of Craniomaxillofacial and Plastic Surgery, Faculty of Dentistry, Alexandria University, Alexandria, Egypt

**Keywords:** Bone defects, Periodontally accelerated osteogenic orthodontics, Osseous grafts, Membrane fixation, Stabilization

## Abstract

**Background:**

Periodontally accelerated osteogenic orthodontics (PAOO) is a treatment for bone defects associated with a lack of bone graft stability, especially in coronal locations. This study aimed to compare a modified technique of membrane fixation that utilizes periosteal sutures (using a pouch design) with the traditional approach, which does not use membrane fixation.

**Methods:**

Twenty-eight patients with a total of 168 teeth treated were divided into two groups: 1-A, in which patients were treated using the modified technique (with membrane fixation), and group 2-B, in which patients were treated using the traditional technique (without membrane fixation). The postoperative bone thickness was evaluated via radiographic examination.

**Results:**

Postoperative improvements in bone augmentation were detected in both groups. At 12 months, the values of the CHBT (measured from the midpoint of the coronal third to the labial cortical surface, 0.84 ± 0.33 mm) and the values of VBL (measured from the alveolar crest to the cemento-enamel junction, − 2.35 ± 0.80 mm)were significantly greater in the modified technique group than those in the traditional technique group (CHBT:0.12 ± 0.21 mm and VBL:-1.39 ± 0.99 mm; *P* = 0.00 and *P* = 0.01).

**Conclusions:**

This study shows that compared to the traditional technique, the modified PAOO technique with membrane fixation using periosteal sutures provides improved graft stabilization, superior coronal augmentation and satisfactory vertical volume.

## Background

Alveolar bone defects, which include dehiscence (a defect that extends to the cervical surface of the root, leading to marginal alveolar bone loss) and fenestration (a window that affects the root surface but is still bordered by bone along its coronal aspect), are challenges in conventional orthodontic treatment [1–3]. These defects can also lead to gingival recession, root exposure and even treatment relapse [4].

In 2001, a new technique known as ‘periodontally accelerated osteogenic orthodontics’ (PAOO) was first introduced by Wilcko et al. [2]. The surgical technique distinguishes itself from traditional orthodontics by a combination of flap design, selective decortication, alveolar augmentation, membrane coverage, and suture closure [5–7]. The PAOO technique, which initiated the regional acceleratory phenomenon, provides both dramatically shorter treatment times and efficient orthodontic tooth movement [2, 4].

However, it remains difficult to achieve sufficient bone formation and to prevent graft migration during the long-term follow-up periods. A previous report by Wang et al. [8] showed that the vertical level of the alveolar bone in the lower incisor region decreased significantly during a 6 months follow-up period. A separate study by Coscia et al. [9] found a reduced bone thickness at coronal sites and significant decrease in the vertical levelsof the alveolar augmentation during decompensation treatment in patients with Class III malocclusion. Although bioabsorbable collagen membranes adapted to cover bone grafts substantially improved tissue regeneration and bone formation [10, 11], the lack of mechanical stability might lead to complications including membrane collapse and graft migration. To solve these problems, methods for membrane fixation have been discussed in a number of studies. For example, Kim et al. [12] utilized temporary skeletal anchorages to achieve the fixation of the absorbable membrane. In his study, a secondary surgery was required to retrieve these fixation materials. More recently, Ma et al. [13] introduced a dumpling technique, in which the graft was fixed to the periosteum for stability. However, none of these approaches enabled predictable bone formation at their target sites. We introduced a modified PAOO technique in a previous study [14], in which a pouch was generated using suture fixation of the membranes to the surrounding periosteum. Over a 1-year follow-up period, favorable results were recorded. This technique could correct the vertically deficiency of the alveolar ridge while maintaining the vertical bone gain.

Therefore, the current study was designed to compare the outcomes of the modified PAOO technique (with membrane fixation using a pouch design) and the conventional PAOO technique (without membrane fixation) using radiography.

## Materials and methods

### Study design

This retrospective cohort study was conducted at the Shanghai Ninth People’s Hospital, Shanghai Jiao Tong University (Shanghai, China) from March 2016 to June 2018. The study was designed in accordance with the tenets of the Declaration of Helsinki for research and the protocol was approved by the ethics committee of the Shanghai Jiao Tong University School of Medicine.

**Fig. 1:**
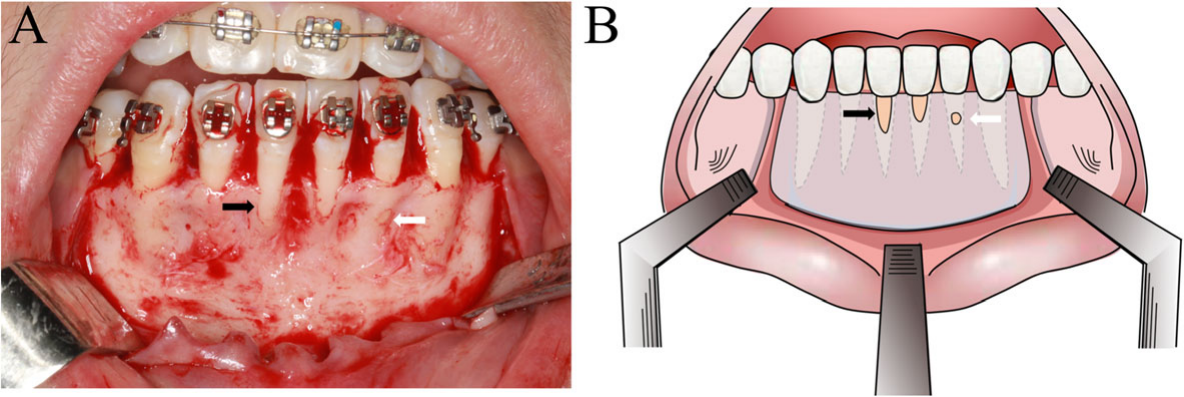
**a** the dehiscence-a vertical root exposed defect where the denuded areas involve the alveolar bone margin (black arrow) and fenestration-a window of bone loss that places the exposed root surface directly in contact with the gingiva or alveolar mucosa (white arrow) on the labial surface of the mandible. **b**: the schematic diagram of dehiscence (black arrow) and fenestration (white arrow)

The inclusion criteria were as follows [14]: 1) patients aged at least 18 years; 2) patients treated with orthodontic camouflage for class II dental malocclusion or a decompensation for class III skeletal malocclusion; and 3) patients with dehiscence (vertical defect exposing the root, with the denuded areas involving the alveolar bone margin) and/or a fenestration (bone loss window placing the exposed root surface in direct contact with the gingiva or the alveolar mucosa) on the labial surface of the mandible (Fig. 1a and b) [13, 15]. Patients were excluded if they were pregnant or lactating, had any sign of periodontitis or systemic diseases (immune suppression, bisphosphonate medication, chemotherapy or radiotherapy, psychological disorders) [9, 13]. Other exclusion criteria included the following: 1)smoking; 2) previous orthodontic or orthognathic treatment; 3) use of any medication that could affect bone metabolism; and 4) inability to complete a 12-months follow-up period [15].

All participants were diagnosed and assessed in parallel by two independent reviewers (XHL and YNZ) who were blinded to the patient’s condition based on the inclusion and exclusion criteria. Discrepancies between the reviewers were resolved through consultation with a third investigator (CY, with over 28 years of experience in oral surgery). The initial patient screening and evaluation were based on a detailed preliminary examination, the study model, and a cone-beam computerized radiographic examination (CBCT) examination (Imaging Sciences International, Hatfield, PA, USA).

**Fig. 2:**
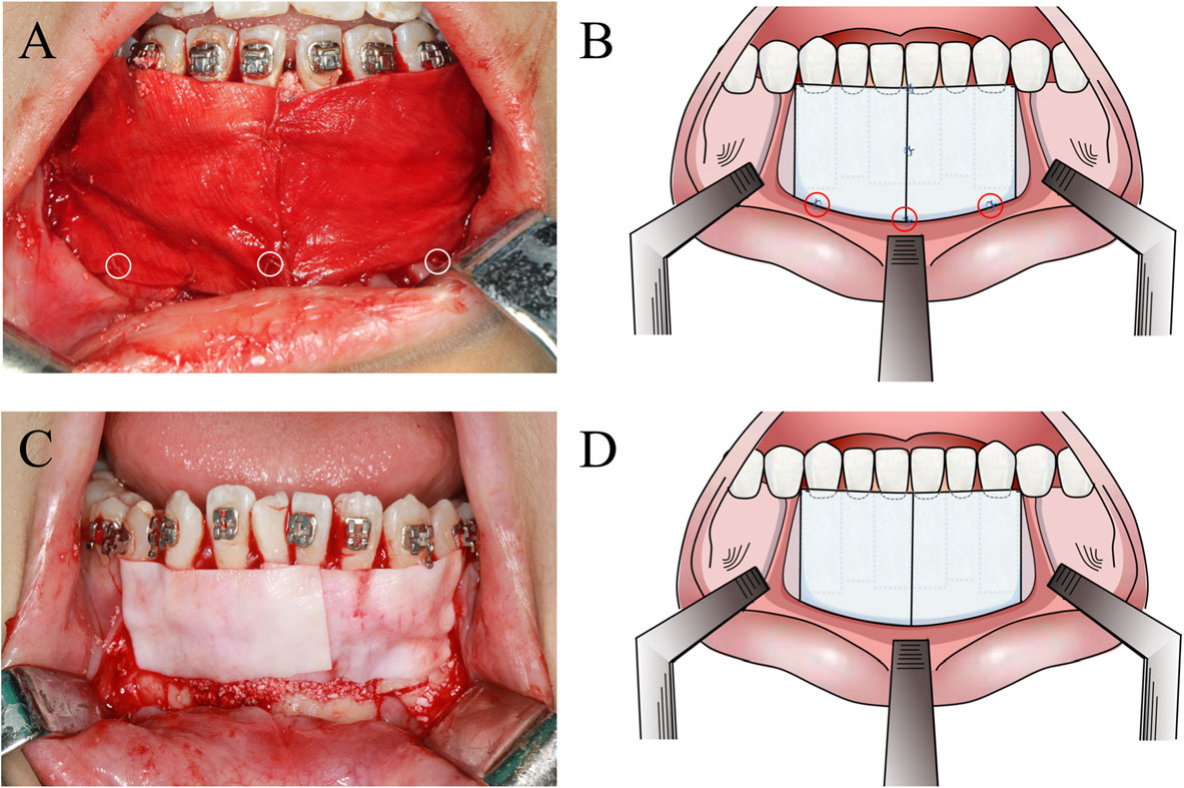
**a** the modified PAOO technique with membrane fixation (using a pouch design)-circles showed the suture; **b**: the schematic diagram for modified group-circles showed the suture; **c**: the traditional PAOO technique without membrane fixation; **d**: the schematic diagram for traditional group

In this study, patients who met the inclusion criteria were assigned to one of two treatment group: group 1-A (Fig. 2), in which the patients were treated by a modified PAOO technique with membrane fixation (using a pouch design); and group 2-B (Fig. 2b), in which the patients were treated by a traditional PAOO technique without membrane fixation.

**Fig. 3:**
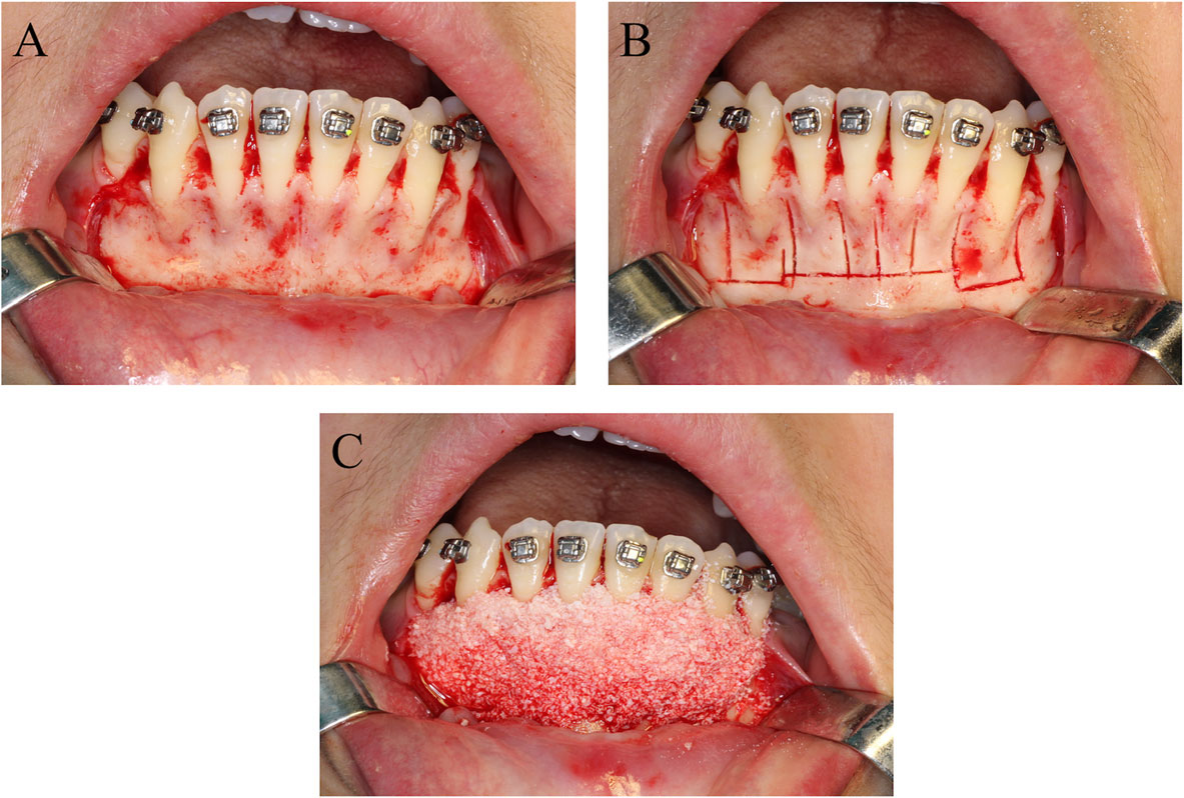
**a** flap with interdental papilla reflected was performed. **b**: the corticotomy which limited to the cancellous bone was performed on the labial aspect of alveolar bone. **c**: the reconstituted deproteinized bovine bone materials mixed with blood collected was subsequently placed into recipient sites

**Fig. 4:**
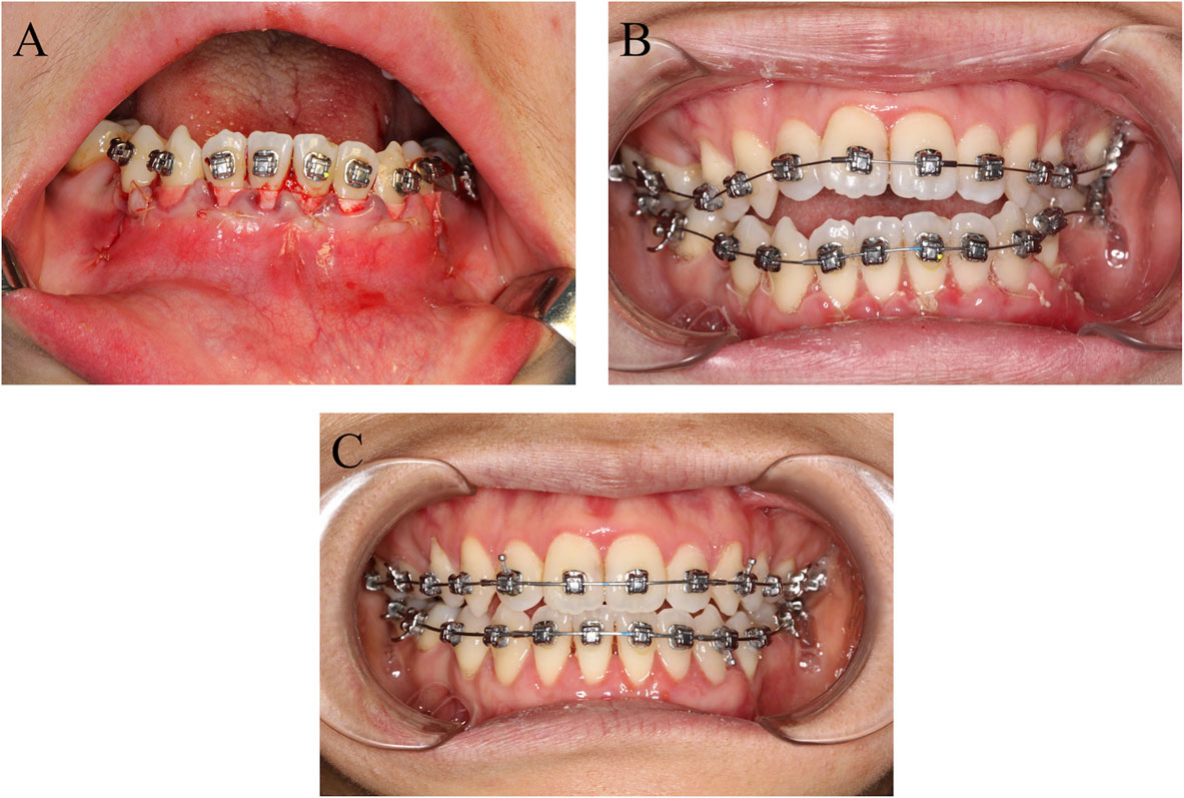
**a** single interrupted sutures were used with 4–0 absorbable polyester, which connected the lingual tissue, the labial flap and the membrane together. **b**: clinical image within the group of flap with interdental papilla reflected in postoperative 1-week.**c**: clinical image within the group of flap with interdental papilla reflected in postoperative 12-months

**Fig. 5:**
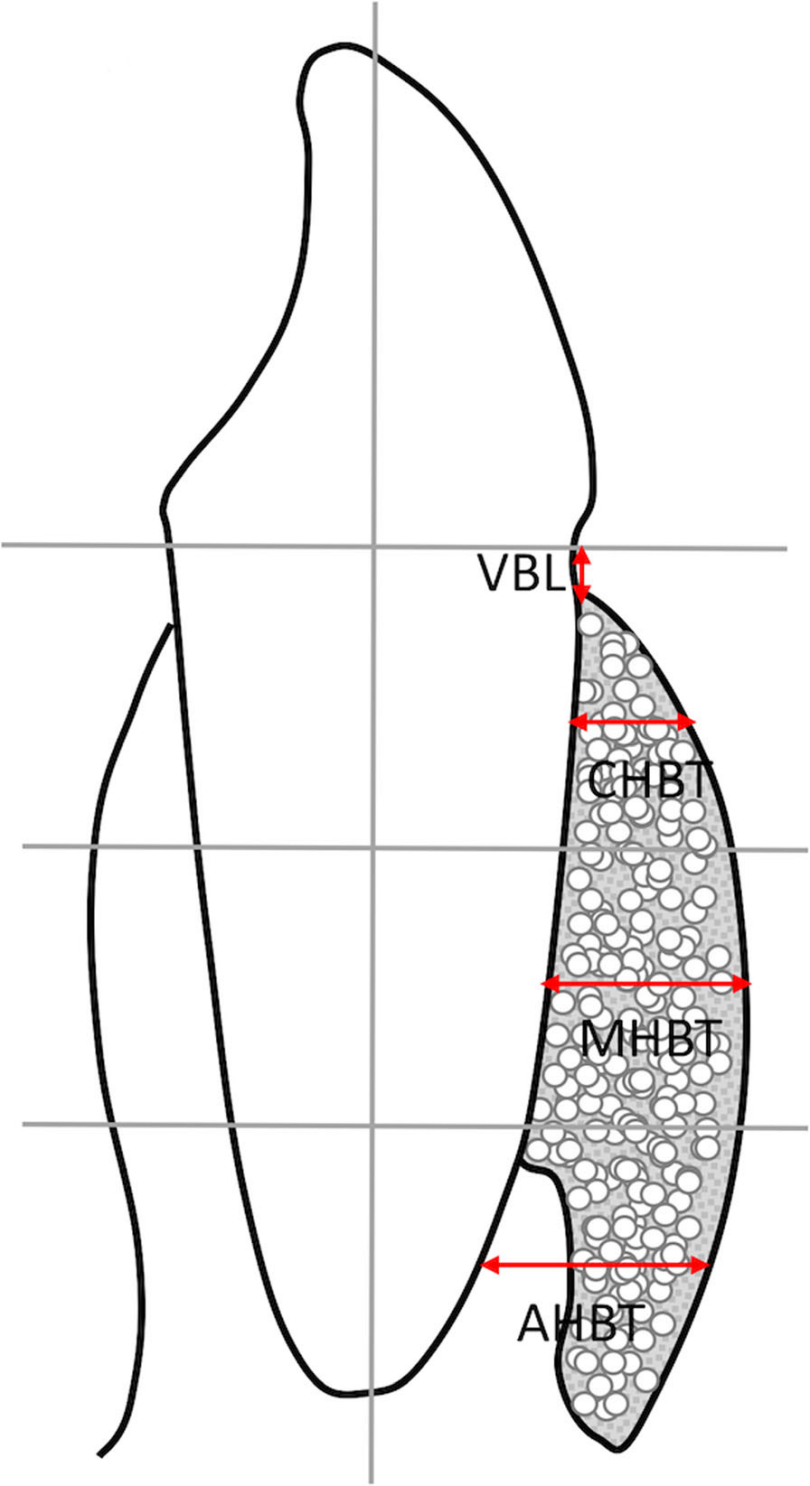
The schematic diagram of radiographic assessments (CHBT, horizontal bone thickness at the middle level of the coronal third; MHBT, horizontal bone thickness at the middle level of the middle third; AHBT, horizontal bone thickness at the middle level of the apical third; VBL, vertical alveolar bone level.)

**Fig. 6:**
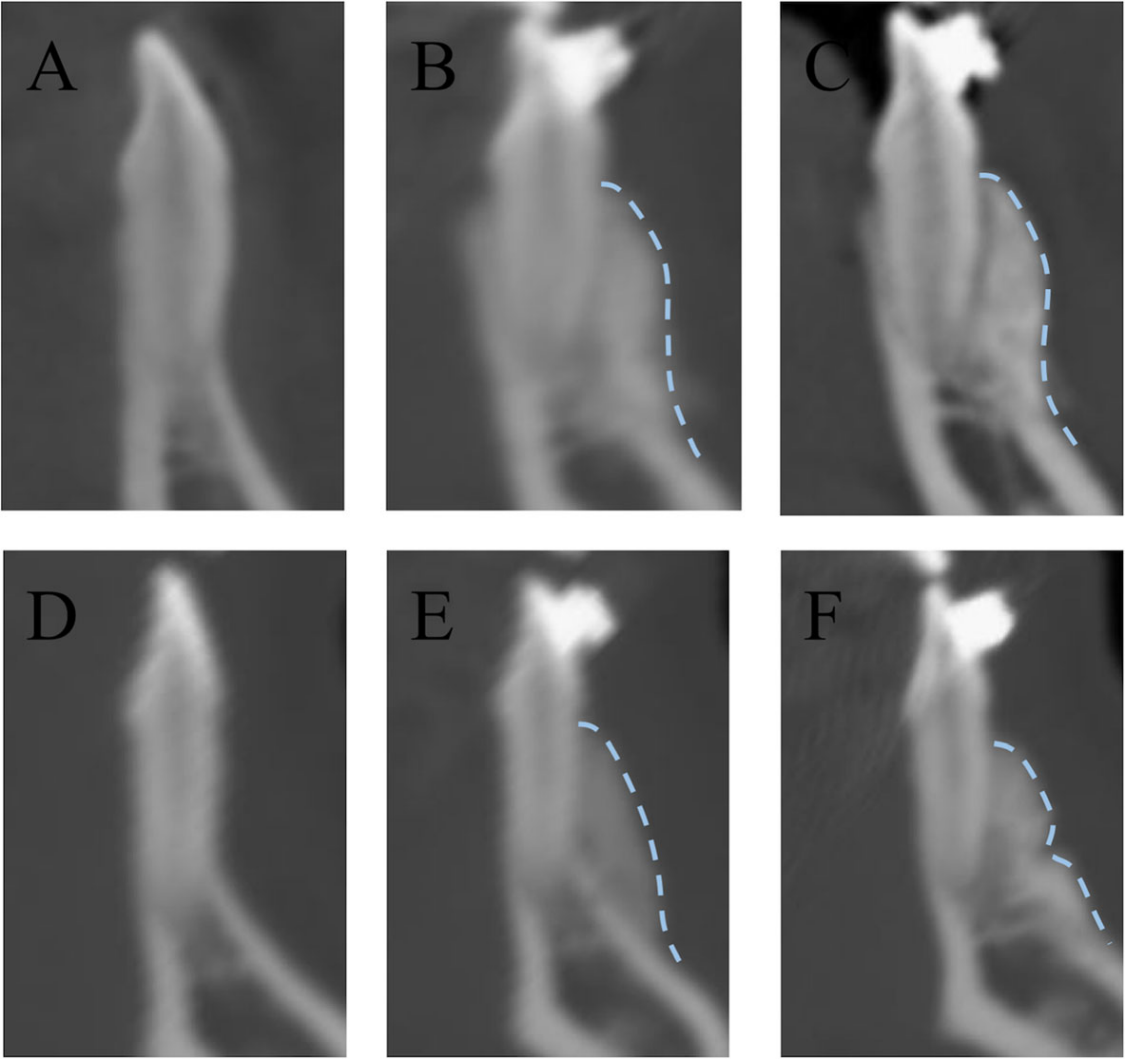
**a** CBCT images in modified group before surgery; **b**: CBCT images in modified group at postoperative 1-week (blue line marked the margin); **c**: CBCT images in modified group at postoperative 12-months (blue line marked the margin); **d**: CBCT images in traditional group before surgery; **e**: CBCT images in traditional group at postoperative 1-week (blue line marked the margin); **f**: CBCT images in traditional group at postoperative 12-months (blue line marked the margin)

### Treatment protocol

In accordance with previous guidelines, each patient was treated with 0.022-  ×  0.028-in. pre-adjusted appliances 1 week prior to surgery under the guidance of the orthodontist [13, 14, 16]. However, orthodontic tooth movement was not performed until 2 weeks after the surgical procedure [17]. Nickel-titanium arch wires (to were aligned and leveled the postoperative arch) and stainless steel arch wires (to complete the treatment) were used in accordance with routine orthodontic adjustment guidelines [8, 9].

### Surgical procedure [14]

The same surgeon (C.Y, with more than 28 years of experience in oral surgery) performed the surgical procedures for all of the patients under local anesthesia (2 to 3 ml of 2% lidocaine) [2, 4].

In both groups, a sulcular releasing incision was first made to create a labial flap with interdental papilla reflection(Fig. 3). The line of this incision was extended by the width of 1 tooth beyond the bone activation area, typically from one distal surface of the canine to the other. To ensure proper flap reflection, a bilateral series of vertical- releasing incisions which extended horizontally was msde in both groups. The flap was then reflected onto the buccal aspect to approximately 3-4 mm beyond the apices of the anterior teeth. Finally, a periosteal releasing incision was made approximately 3–4 mm apical to the mucogingival junction using surgical scissors. To avoid damage to the mental neurovascular bundles, the coronal depth of the membrane release was limited to 3 mm.

For selective decortication (Fig. 3b), a corticotomy that was limited to the cancellous bone was performed using a piezoelectric surgical device (Piezosurgery, Silfragent, Italy) on the labial aspect of the alveolar bone. The vertical grooves, which were placed at the midpoint of the interradicular space, typically started 1–2 mm below the alveolar crest and extended to a point that was 2–3 mm beyond the apices of the roots where the horizontal groove was placed.

Following the corticotomy procedure, reconstituted deproteinized bovine bone (Bio-Oss, Geistlich Biomaterials AG, Wolhusern, Switzerland) mixed with blood collected from surgical area (an estimated volume of 0.3 ml per tooth) was placed into the recipient sites (Fig. 3c) [9].

Following the graft placement, two bioabsorbable collagen membranes for guided tissue (Bio-Guide, 25  ×  25 mm, Geistlich Biomaterials AG) were placed directly over the graft material in the traditional group. However, in the modified group, two collagen membranes were first sutured together to prevent mutual shifting. Then, the combined collagen membrane was suturedto the lower periosteum and placed over the bone material that was positioned in the prepared pouch [11]. The suturing process involved 3 punctures that were initiated on the distal aspect, followed by the middle and mesial aspects. Suturing was performed with an FS-2 needle and a 4–0 absorbable sutures (Biosyn, Covidien, Mansfield, MA) by penetrating the periosteum at the coronal aspect toward the periosteal releasing incision and then passing through the membrane and placing a simple surgeon’s knot.

After ensuring that the membrane was positioned over the graft, the buccal flap was repositioned in both groups and sutured with a 4–0 synthetic nonabsorbable polyester. In the modified group, single interrupted sutures were used to connect the lingual tissue, the labial flap and the membrane (Fig. 4).

In both group, pressure was provided with a chin cap for at least 7 days to ameliorate postoperative swelling or edema and maintain graft stability. Routine antibiotics, nonsteroidal anti-inflammatory drugs, and analgesic agents were prescribed for use for at least 3 days, and the use of a 0.12% chlorhexidine gluconate solution for 1 min, twice a day for 2 consecutive weeks was recommended. Patients were asked to participate in regular followed-up to receive clinical and radiographic evaluations (Fig. 4b and c).

### Radiographic parameters for augmented bone quantification

Radiographic measurements were obtained by standardized CBCT using an iCAT scanner (VG; NewTom, Verona, Italy) in a single 360^o^ rotation with a 20-s scan time at 110 kV, 0 to 20 mA and a 0.125-mm voxel size. Digital files of the heaviest section from the 3 spatial planes (coronal, horizontal, and sagittal) obtained were adjusted to be perpendicularly to the long axis of each anterior tooth being tested [9, 13, 14] (Fig. 5) for determination of the following parameters: 1) CHBT, measured from the midpoint of the coronal third to the labial cortical surface; 2) MHBT, measured from the midpoint of the middle third to the labial cortical surface; 3) AHBT, measured from the midpoint of the apical third to the labial cortical surface; and 4) VBL, measured from the alveolar crest to the cemento-enamel junction.

The radiographic measurements at different time points (T0: before surgery; T1: 1 week; and T2: 12 months after surgery) were made by a third operator (XHL) who was unaware of the patient’s assignment (Fig. 6).

### Statistical analysis

The collected data were analyzed using the Statistical Package for Social Sciences (SPSS, version 12.0, Chicago, IL). The descriptive statistics were based on the mean of two measurements, were rounded up to the nearest millimeter and were presented as the means ± SD. Differences between the two groups were analyzed using a two-sample independent t-test. Intra- and intergroup differences were analyzed using the intraclass correlation coefficients (ICCs). Statistically significant differences were considered for a probability values less than 0.05 (*P* < 0.05).

## Results

From March 2016 to June 2018, 28 patients(168 treated teeth) who met the inclusion criteria were recruited into this study. Of these patients, 14 patients (84 sites) were assigned to the modified PAOO group (1-A), in which used membrane fixation was used, and 14 patients (84 sites) were assigned to the traditional PAOO group (2-B), in which membrane fixation was not used. No patients dropped out of this 12-month study. The ICCs for intra-r and interobserver agreement ranged from 0.94 to 0.98 and 0.80 to 0.95, respectively, indicating excellent reliability.

### Evaluation of augmented bone quantity

In both groups (Table 1), postoperative improvements compared to the baseline was detected. At week 1 postoperatively, no statistically significant differences in the graft material measurements were observed between the groups (CHBT: *P* = 0.98; MHBT: *P* = 0.75; AHBT: *P* = 0.69; VBL: *P* = 0.58). However, by 12 months postoperatively, the CHBT was significantly higher in the modified group than in the traditional group (0.84 ± 0.33 mm in group 1-A vs. 0.12 ± 0.21 mm in group 2-B group, *P* = 0.00). Additionally, the change in the VBL was significantly greater in group 1-A than in group 2-B (− 2.35 ± 0.80 vs − 1.39 ± 0.99, *P* = 0.01). In contrast, there was no statistically significant differences between the groups in the MHBT or AHBT (*P* = 0.62, *P* = 0.14).

**Table 1:** The comparison of data between groups^a^

Parameter, mm			Traditional group (*n* = 84)	Modified group (n = 84)	*P* value
∆Bone thickness^§^	∆CHBT	Baseline	0.34 ± 0.19	0.37 ± 0.15	/
		T1-T0^†^	1.40 ± 0.18	1.41 ± 0.28	0.98
		T2-T0^†^	0.12 ± 0.21	0.84 ± 0.33	0.00*
	∆MHBT	Baseline	0.73 ± 0.53	0.59 ± 0.17	/
		T1-T0^†^	2.62 ± 0.62	2.68 ± 0.19	0.75
		T2-T0^†^	1.60 ± 0.54	1.69 ± 0.37	0.62
	∆AHBT	Baseline	1.77 ± 0.75	1.63 ± 0.17	/
		T1-T0^†^	2.43 ± 0.59	2.52 ± 0.63	0.69
		T2-T0^†^	1.69 ± 0.43	1.46 ± 0.39	0.14
	∆VBL	Baseline	4.32 ± 1.42	4.27 ± 0.98	/
		T1-T0^†^	−3.02 ± 0.88	−2.95 ± 0.78	0.58
		T2-T0^†^	−1.39 ± 0.99	−2.35 ± 0.80	0.01*

^a^ Two-samples independent t-test and test for normality was significant (P < 0.05); Plus–minus values are means ±SD

§ CHBT: from the midpoint of the coronal third to the labial cortical surface; MHBT: from the midpoint of the middle third to the labial cortical surface; AHBT: from the midpoint of the apical third to the labial cortical surface; VBL: from the alveolar crest to the cementoenamel junction

† T1-T0:1-week to baseline; T2-T0:12-months to baseline

## Discussion

Alveolar bone defects, which include dehiscence and fenestration, are common complications following orthodontic treatment and can result in root exposure, gingival recession, and treatment relapse [1]. The corticotomy procedure with alveolar augmentation, which combines flap design, selective alveolar corticotomy, particulate bone grafting, absorbable membranes, suture closure and postoperative orthodontic treatment, has been used to treat bone recession and has been gradually recognized for its improved outcomes in terms of alveolar bone thickness, posttreatment stability, and reduced treatment duration [2–4].

However, graft migration, membrane collapse, and insufficient bone formation, remains challenges for bone augmentation during the long-term follow-up period.

Lee et al. [6] reported that there was reduced regeneration in the vertical alveolar augmentation following surgery. Later, another study showed reduced bone thickness at the coronal site in patients with class III malocclusion after treatment [2]. Wang et al. [8] reported on eight consecutive patients with class III malocclusion who were treated with augmented corticotomy-assisted surgical orthodontics. The treatment provided accelerated orthodontic forces and adequate decompensation of the lower incisors, but the regenerative effect of the alveolar augmentation appeared to be concentrated in the middle and apical regions when compared with the coronal level during the 6-months follow-up period [9]. Similarly, the CHBT value reached by the graft material in the traditional group in our study increased by 1.40 mm at 1 week postoperatively and then gradually decreased over the 12-months follow-up period. The mean VBL was 1.3 mm at 1-week postoperatively and 2.93 mm at 12-months postoperatively (*P* < 0.05).

Bioabsorbable collagen membranes, which are associated with excellent results in tissue regeneration and bone formation, can always be adapted to cover the grafting sites. However, due to the lack of mechanical stability, movement of the membrane can lead to membrane collapse and graft migration. Shalev et al. [10] showed that external pressure from the flap or occlusal forces can laterally and apically displace the membrane, resulting in a deficient bone formation in the desired region. Therefore, it is essential to stabilize the absorbable membrane to achieve particulate graft-induced formation in the predicted region. Kim et al.’s [12] reported a different technique involving an absorbable membrane with a temporary skeletal anchor. Although rigid scaffolding materials can prevent membrane migration and facilitate bone formation at the coronal aspect of the root, wound dehiscence, titanium plate explosure and the need for secondary retrieval surgery can also occur.

Recently, we introduced a modified PAOO technique to generated a pouch by fixing the membranes to the surrounding periosteum with sutures [14]. Over the 1-year follow-up period, favorable results were found and demonstrated the ability of the technique to correct the vertically deficientcy of the alveolar ridge while maintaining the vertical bone gain. Therefore, the current study was designed to evaluate the outcomes of the modified PAOO technique by comparing it with the conventional technique.

Urban et al. [18] showed that the tensile strength of the absorbable suture materials decreased over time, which might have a negative effect on the membrane fixation and graft stabilization. While there have been no reports on the time required for membrane fixation, previous studies have shown that a preliminary bone matrix is established after the initial weeks of healing [19]. In the current study, an absorbable collagen membrane was fixed to the lower periosteum incision with three simple interrupted sutures to achieved a proper apicocoronal graft fixation. The CHBT and VBL values increased by 0.84 mm and 2.35 mm, respectively, and these increases were much higher than those observed in the control group (0.12 mm and 1.39 mm, respectively), during the 12-month follow-up period.

In previous study, Ma et al. [13] developed a dumpling technique, in which the traditional bioabsorbable collagen membrane was replaced with the periosteum. However, it was still challenging to use an incision at the mucogingival junction for the placement of particulate grafting material into cervical defects. Moreover, the periosteal dissection method in the technique is complex, requires a long operation time, and carries a greater risk of nerve injury. Additionally, the lack of sufficient periosteal support can result in direct communication between the bone graft and the oral cavity, which can lead to complications, such as grafting material leakage and necrosis. In contrast, the fixed membrane in our study provides full bone graft coverage while maintaining the graft components in a more compacted state, which provides a favorable environment for alveolar augmentation.

The limitations of this study must also be considered. Due to the small sample size, the subjects were not divided into blocks to account for age or sex. Future studies should also utilize a randomized block design. Additionally, because our study mainly focused on measuring the quantity of new bone formation, future studies should also evaluate the quality of the newly formed bone.

## Conclusions

The results of this comparative study demonstrate that the modified PAOO technique, in which a pouch is created by fixation of the membranes to the surrounding periosteum using sutures, provides adequate graft stabilization with superior coronal augmentation and satisfactory vertical volume.

## Data Availability

The datasets used and/or analysed during the current study available from the corresponding author on reasonable request.

## Abbreviations

AHBT: From the midpoint of the apical third to the labial cortical surface
CBCT: Cone-beam computerized tomography
CHBT: From the midpoint of the coronal third to the labial cortical surface
MHBT: From the midpoint of the middle third to the labial cortical surface
PAOO: Periodontally accelerated osteogenic orthodontics
VBL: From the alveolar crest to the cementoenamel junction

## References

[1] Dahlin C, Lekholm U, Becker W, Becker B, Higuchi K, Callens A (1995). Treatment of fenestration and dehiscence bone defects around oral implants using the guided tissue regeneration technique: a prospective multicenter study. Int J Oral Maxillofac Implants.

[2] Wilcko WM, Wilcko T, Bouquot JE, Ferguson DJ (2001). Rapid orthodontics with alveolar reshaping: two case reports of decrowding. Int J Periodontics Restorative Dent.

[3] Yagci A, Veli I, Uysal T, Ucar FI, Ozer T, Enhos S (2012). Dehiscence and fenestration in skeletal class I, II, and III malocclusions assessed with cone-beam computed tomography. Angle Orthod.

[4] Wilcko MT, Wilcko WM, Pulver JJ, Bissada NF, Bouquot JE (2009). Accelerated osteogenic orthodontics technique: a 1-stage surgically facilitated rapid orthodontic technique with alveolar augmentation. J Oral Maxillofac Surg.

[5] Wennstrom JL (1996). Mucogingival therapy. Ann Periodontol.

[6] Lee KM, Kim YI, Park SB, Son WS (2012). Alveolar bone loss around lower incisors during surgical orthodontic treatment in mandibular prognathism. Angle Orthod.

[7] Murphy KG, Wilcko MT, Wilcko WM, Ferguson DJ (2009). Periodontal accelerated osteogenic orthodontics: a description of the surgical technique. J Oral Maxillofac Surg.

[8] Wang B, Shen G, Fang B, Yu H, Wu Y, Sun L (2014). Augmented corticotomy-assisted surgical orthodontics decompensates lower incisors in class III malocclusion patients. J Oral Maxillofac Surg.

[9] Coscia G, Coscia V, Peluso V, Addabbo F (2013). Augmented corticotomy combined with accelerated orthodontic forces in class III orthognathic patients: morphologic aspects of the mandibular anterior ridge with cone-beam computed tomography. J Oral Maxillofac Surg.

[10] Shalev TH, Kurtzman GM, Shalev AH, Johnson DK, Kersten MEM (2017). Continuous periosteal strapping sutures for stabilization of osseous grafts with Resorbable membranes for buccal ridge augmentation: a technique report. J Oral Implantol.

[11] Stoecklin-Wasmer C, Rutjes AW, da Costa BR, Salvi GE, Juni P, Sculean A (2013). Absorbable collagen membranes for periodontal regeneration: a systematic review. J Dent Res.

[12] Kim SH, Kim I, Jeong DM, Chung KR, Zadeh H (2011). Corticotomy-assisted decompensation for augmentation of the mandibular anterior ridge. Am J Orthod Dentofac Orthop.

[13] Ma ZG, Yang C, Xie QY, Ye ZX, Zhang SY, Abdelrehem A (2016). A novel surgical technique for augmented Corticotomy-assisted orthodontics: bone grafting with periosteum. J Oral Maxillofac Surg.

[14] Ma Z, Zheng J, Yang C, Xie Q, Liu X, Abdelrehem A (2018). A new modified bone grafting technique for periodontally accelerated osteogenic orthodontics. Medicine (Baltimore).

[15] Ge J, Yang C, Zheng J, Hu Y (2017). Autogenous bone grafting for treatment of osseous defect after impacted mandibular third molar extraction: a randomized controlled trial. Clin Implant Dent Relat Res.

[16] Chung KR, Oh MY, Ko SJ (2001). Corticotomy-assisted orthodontics. J Clin Orthod.

[17] Amit G, Jps K, Pankaj B, Suchinder S, Parul B (2012). Periodontally accelerated osteogenic orthodontics (PAOO) - a review. J Clin Exp Dent.

[18] Urban IA, Lozada JL, Wessing B (2016). Suarez-Lopez del Amo F, Wang HL. Vertical bone grafting and periosteal vertical mattress suture for the fixation of Resorbable membranes and stabilization of particulate grafts in horizontal guided bone regeneration to achieve more predictable results: a technical report. Int J Periodontics Restorative Dent.

[19] Tonetti M, Palmer R (2012). Working group 2 of the VEWoP. Clinical research in implant dentistry: study design, reporting and outcome measurements: consensus report of working group 2 of the VIII European workshop on periodontology. J Clin Periodontol.

